# Chloride imbalance in Fragile X syndrome

**DOI:** 10.3389/fnins.2022.1008393

**Published:** 2022-10-12

**Authors:** Kaleb Dee Miles, Caleb Andrew Doll

**Affiliations:** Department of Pediatrics, Section of Developmental Biology, University of Colorado School of Medicine, Children’s Hospital Colorado, Aurora, CO, United States

**Keywords:** fragile X syndrome, GABA, chloride transporters, inhibition, excitation

## Abstract

Developmental changes in ionic balance are associated with crucial hallmarks in neural circuit formation, including changes in excitation and inhibition, neurogenesis, and synaptogenesis. Neuronal excitability is largely mediated by ionic concentrations inside and outside of the cell, and chloride (Cl^–^) ions are highly influential in early neurodevelopmental events. For example, γ-aminobutyric acid (GABA) is the main inhibitory neurotransmitter of the mature central nervous system (CNS). However, during early development GABA can depolarize target neurons, and GABAergic depolarization is implicated in crucial neurodevelopmental processes. This developmental shift of GABAergic neurotransmission from depolarizing to hyperpolarizing output is induced by changes in Cl^–^ gradients, which are generated by the relative expression of Cl^–^ transporters Nkcc1 and Kcc2. Interestingly, the GABA polarity shift is delayed in Fragile X syndrome (FXS) models; FXS is one of the most common heritable neurodevelopmental disorders. The RNA binding protein FMRP, encoded by the gene Fragile X Messenger Ribonucleoprotein-1 (Fmr1) and absent in FXS, appears to regulate chloride transporter expression. This could dramatically influence FXS phenotypes, as the syndrome is hypothesized to be rooted in defects in neural circuit development and imbalanced excitatory/inhibitory (E/I) neurotransmission. In this perspective, we summarize canonical Cl^–^ transporter expression and investigate altered gene and protein expression of Nkcc1 and Kcc2 in FXS models. We then discuss interactions between Cl^–^ transporters and neurotransmission complexes, and how these links could cause imbalances in inhibitory neurotransmission that may alter mature circuits. Finally, we highlight current therapeutic strategies and promising new directions in targeting Cl^–^ transporter expression in FXS patients.

## Introduction

Fragile X syndrome (FXS) is one of the most common heritable neurodevelopmental disorders, which is characterized by intellectual disability, epilepsy, and behavioral symptoms such as anxiety and hyperactivity (Bhakar et al., 2012; Nelson et al., 2013). The etiology stems from a trinucleotide expansion mutation in the 5′ untranslated region of the FMR1 gene, thus leading to the loss of fragile X messenger ribonucleoprotein (FMRP) (Verkerk et al., 1991). FMRP plays essential roles in a variety of neurodevelopmental processes, including synaptogenesis, cell fate specification, and differentiation (Lu et al., 2004; Antar et al., 2006; Doll et al., 2021). Canonically, FMRP functions as an RNA binding protein (RBP), regulating the expression of an array of mRNAs associated with autism spectrum disorders and synaptic processes (Darnell et al., 2011; Ascano et al., 2012). It is estimated that 1 in 7,000 males and 1 in 11,000 females have FXS, and approximately 25–33% of individuals with FXS also meet the criteria for autism spectrum disorders (ASD) by displaying strong overlap in behavioral symptoms (Hunter et al., 2014; Kaufmann et al., 2017). With the rising prevalence and complex nature of these disorders, researchers are faced with many challenges to decipher the pathophysiology and develop therapeutics (Newschaffer et al., 2005; Contractor et al., 2021).

An established hallmark of FXS is hyperactivity, which has been associated with imbalanced excitation and inhibition (E/I ratios) in neural circuits. As the major inhibitory neurotransmitter in the brain, GABAergic influence on FXS pathogenesis represents a key component of this theory. Indeed, there is evidence that reduced GABAergic output in FXS results in hypoinhibition and imbalanced E/I output (Gibson et al., 2008; Olmos-Serrano et al., 2010; Goncalves et al., 2013). However, motor circuits begin to form in embryogenesis, and neurotransmission in embryonic stages is distinct from the mature nervous system. For example, in early neurodevelopment the main “inhibitory” neurotransmitters, GABA and glycine, can depolarize receptive neurons (Zhang et al., 2006, 2010; Reynolds et al., 2008). This excitatory influence on immature neurons is associated with crucial neurodevelopmental processes, including neural stem cell proliferation, cell migration, neurite outgrowth, synapse formation, and network oscillations, all of which represent critical hallmarks in neural circuit formation (Behar et al., 2000; Liu et al., 2005; Akerman and Cline, 2006; Ben-Ari et al., 2007; Cancedda et al., 2007; Wang and Kriegstein, 2008). As development ensues, GABA reception undergoes a polarity shift from excitatory to inhibitory output, which is dictated by the relative expression of chloride ion (Cl^–^) transporters and Cl^–^ dynamics (Rivera et al., 1999; Yamada et al., 2004; Watanabe and Fukuda, 2015). However, it has been shown in models of FXS, ASD, and other neurodevelopmental disorders that this polarity shift is disrupted or delayed (Talos et al., 2012; Duarte et al., 2013; He et al., 2014; Tyzio et al., 2014; Banerjee et al., 2016; Ruffolo et al., 2016; Tang et al., 2016; Amin et al., 2017; Li et al., 2017; Hinz et al., 2019). Importantly, persistent depolarization by GABA and glycine could influence epileptic and repetitive behaviors observed in these disorders (Duarte et al., 2013; He et al., 2014; Tyzio et al., 2014; Banerjee et al., 2016; Tang et al., 2016; Huo et al., 2017; Hinz et al., 2019).

Researchers have attempted to restore excitatory/inhibitory (E/I) balance in FXS and ASD by manipulating GABAergic and glutamatergic (Glu) neurotransmission. Collectively, animal studies for both GABA and Glu modulators have produced promising results by promoting proper dendritic spine development, reducing severity of seizures, and ameliorating repetitive behaviors (He et al., 2002; Silverman et al., 2010; Henderson et al., 2012; Wei et al., 2012). Subsequent GABA modulator clinical trials assessing acamprosate, arbaclofen, riluzole/risperidone, and valproate have collectively shown improved social behavior, hyperactivity, communication skills, compulsive symptoms, and irritability in pediatric and some young adult subjects (Hellings et al., 2005; Hollander et al., 2006; Erickson et al., 2010, 2011a,b; Berry-Kravis et al., 2012). Other approaches have targeted Cl^–^ transporters (Nkcc1 and Kcc2) by selectively inhibiting Nkcc1 function or enhancing Kcc2 expression to restore the E/I balance (Guida et al., 2015; He et al., 2019; Tang et al., 2019; Urbanska et al., 2019). Although we lack the space to review mechanisms of Cl^–^ transporter expression, previous reviews have detailed Cl^–^ transporter transcription, post-translational modifications, and function (Watanabe and Fukuda, 2015; Schulte et al., 2018); these studies have contributed to the development of new therapeutic strategies and represent a true success story in the interplay between basic science and the clinic. We also recognize a few of the excellent reviews on GABA and chloride transporter associations to neurodevelopmental disorders (Paluszkiewicz et al., 2011; Schulte et al., 2018; Liu et al., 2022).

This is a developmental perspective on chloride transporter links to FXS and associated neurodevelopmental disorders. We also address the development of spinal networks, which may be especially pertinent to the field as FXS, ASDs, and NDDs are all associated with motor challenges. We first summarize the canonical Cl^–^ transporter expression and function in typical neurodevelopment and in FXS. We then discuss roles for depolarizing GABAergic signaling in critical developmental processes. Next, we present non-canonical roles for Cl^–^ transporters, which could also influence inhibitory neurotransmission. Finally, we highlight the current developments and advancements that establish Cl^–^ transporters as therapeutic targets. It is important to note that our primary focus is on GABAergic signaling, though glycinergic transmission—most predominant in the spinal cord—is also depolarizing in immature neurons (Wu et al., 1992; Rivera et al., 1999).

## Chloride transporter expression underlies the GABAergic polarity shift

**FIGURE 1:**
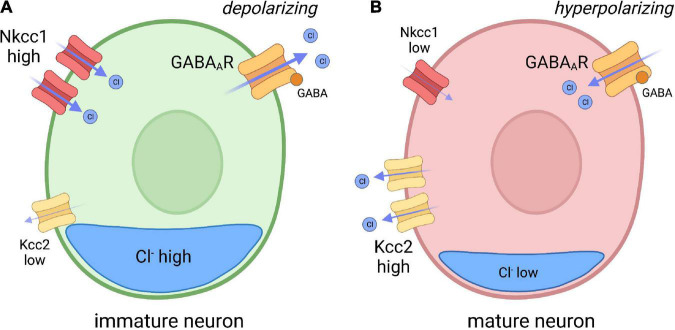
Model depicting chloride (CI) transporter expression in immature and mature neurons. **(A)** In immature neurons, the Nkcc1 Cl^–^ importer is highly expressed, while the Kcc2 Cl^–^ exporter expression is low, resulting in high internal chloride concentration. As GABA A receptors (GABA_*A*_R) are Cl^–^ permeable, GABA reception spurs depolarization of the membrane through Cl^–^ efflux. **(B)** In mature neurons, Kcc2 expression is increased, resulting in reduced internal Cl^–^ concentration compared to immature neurons. GABA binding to GABA_*A*_R in mature neurons therefore drives hyperpolarization. Inspired by Ben-Ari, Trends in Neurosciences, 2017.

Inhibitory neurotransmission is largely dependent on electrochemical ion gradients established at the plasma membrane of neurons receptive to GABAergic and glycinergic signaling. The primary inhibitory receptors of the CNS, GABA_*A*_ receptors and glycinergic receptors, are ionotropic anion permeable channels that allow the passage of chloride (Cl^–^) across the plasma membrane (Kaila, 1994; Kirsch, 2006). The Cl^–^ gradient is mainly established by two Cl^–^ transporters: Nkcc1, a Cl^–^ importer; and Kcc2, a Cl^–^ exporter. Therefore, the expression and activity of both transporters influences intracellular Cl^–^ concentration, which ultimately determines the polarity of GABAergic and glycinergic transmission. In immature neurons, Nkcc1 expression is greater than Kcc2, resulting in a higher intracellular concentration of Cl^–^ (Rivera et al., 1999). As GABA receptors are Cl^–^ permeable, GABA signaling can depolarize immature neurons (reflecting Cl^–^ efflux) resulting in a higher propensity toward neuronal excitation (Kaila, 1994; Dzhala et al., 2005; Moore et al., 2017). In contrast, increased Kcc2 function lowers intracellular Cl^–^ content in more mature neurons, which now receive GABAergic signaling in a hyperpolarizing (inhibitory) manner (Figure 1). The developmental upregulation of Kcc2 expression is a conserved process, as described in humans, rats, mice, zebrafish, and C. *elegans* (Gulyás et al., 2001; Hübner et al., 2001; Tanis et al., 2009; Zhang et al., 2010; Huo et al., 2017). The polarity-switch is crucial for the formation of inhibitory networks that limit excitability in the nervous system.

Chloride transporter expression is regulated by regional and temporal mechanisms that appear to coincide with the maturation of distinct neural networks. In the rodent brain, Nkcc1 is expressed in the precursors of the neuroepithelium, while Kcc2 is primarily expressed in differentiated neurons (Li et al., 2002; Wang et al., 2002; Stein et al., 2004). Kcc2 expression also correlates with the progressive maturation of different brain regions, as it is first expressed in the caudal spinal cord and medulla and then proceeds to more rostral structures in the brain (Li et al., 2002; Stein et al., 2004; Watanabe and Fukuda, 2015). Interestingly, the reversal potential of GABA type A receptor- (GABA_*A*_R-) mediated currents varies among neuronal populations and brain structures, which reflects the diverse expression and function of the transporters (Watanabe and Fukuda, 2015). For example, Kcc2 is expressed in motor neurons during early embryonic stages when GABAergic/glycinergic neurotransmission is still depolarizing (Hübner et al., 2001; Delpy et al., 2008). This may suggest that Nkcc1-mediated chloride import is more predominant at this stage, prior to the inhibitory influence of GABA in more mature stages.

Changes in chloride transporter expression and function are also associated with neurodevelopmental disorders that share common patient phenotypes, most notably epilepsy. The GABA polarity shift is delayed in FXS and other neurodevelopmental disorders, and there is evidence that dysregulation of the Cl^–^ transporters may underlie altered inhibitory transmission (Talos et al., 2012; He et al., 2014; Banerjee et al., 2016; Amin et al., 2017). In Fmr1 knockout mice, Nkcc1 protein expression is upregulated in the cortex at P10, which may account for altered chloride homeostasis and delayed polarity shift in these animals (He et al., 2014). In addition, inhibition of Nkcc1 function *via* bumetanide during the critical period of somatosensory development restores sensory deficits and neuronal morphology in Fmr1 knockout mice (He et al., 2019). Although it is unclear if FMRP directly regulates the translation of Nkcc1 and Kcc2, FMRP binds the transcripts encoding these proteins, *Slc12a2* and *Slc12a5*, respectively (Darnell et al., 2011). Intriguingly, mutations in *SLC12A2* (encoding NKCC1) are linked with neurodevelopmental disorders, and genetic variation in *SLC12A5* (encoding KCC2) is associated with epilepsy and autism (Kahle et al., 2014; Puskarjov et al., 2014; Merner et al., 2015; McNeill et al., 2020; Stödberg et al., 2020).

Chloride transporter misexpression is also noted in related neurodevelopmental disorders. Some patients with tuberous sclerosis (a condition often co-morbid with seizures) show upregulated Nkcc1 and downregulated Kcc2 in cortical tubers (Talos et al., 2012; Ruffolo et al., 2016). Hippocampal neurons from DiGeorge syndrome model mice also show increased expression of Nkcc1 and reduced expression of Kcc2 (Amin et al., 2017). Kcc2 downregulation is also observed in VPA-induced rodent models of autism and neurons derived from patients with Rett syndrome (Duarte et al., 2013; Banerjee et al., 2016; Tang et al., 2016; Hinz et al., 2019). Finally, both rodent and human temporal lobe epilepsy studies show upregulated Nkcc1 and downregulated Kcc2 (Karlócai et al., 2016; Auer et al., 2020; Hampel et al., 2021). These findings are especially important as these neurodevelopment disorders share a common comorbidity of increased seizure susceptibility, which is speculated to be a cortical manifestation of runaway hyperexcitability produced by E/I imbalance (Canitano, 2007; González and Bautista, 2009; Glaze et al., 2010; He et al., 2014; Ruffolo et al., 2016). Taken together, many neurodevelopmental disorders that share common hyperexcitable phenotypes are associated with misregulated chloride transporter expression.

## The influence of depolarizing GABA and glycine on neurodevelopment

### Cell proliferation and synaptogenesis

**FIGURE 2:**
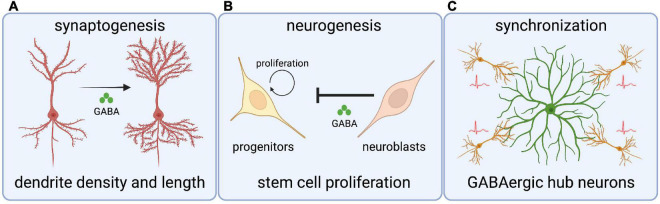
Roles for chloride transporters and depolarizing GABA in crucial neurodevelopmental processes. **(A)** Depolarizing GABA (through Cl^–^ transporters) regulates dendritic spine length and density in cortical neurons. **(B)** GABA signaling from neuroblasts inhibits stem cell proliferation in the subventricular zone. **(C)** GABAergic hub interneurons synchronize nascent hippocampal networks through giant depolarizing potentials.

The GABAergic influence on neurodevelopment begins with roles in regulating neurogenesis (Figure 2). GABA can act as a cell cycle inhibitor in neural precursors, organotypic brain slices, and in the subventricular zone (SVZ) of adult mice (Nguyen et al., 2003; Liu et al., 2005). Immature neuroblasts in the SVZ express non-synaptic GABA that drives depolarizing GABA_*A*_R reception in GFAP-expressing progenitor cells, which ultimately leads to a reduction in the number of proliferative stem cells (Liu et al., 2005). This shows that newly generated GABAergic neurons restrict neurogenesis by inhibiting stem cell proliferation (Liu et al., 2005; Platel et al., 2008). Moreover, premature reversal of the chloride gradient through global overexpression of Kcc2 in zebrafish hinders the production of spinal neurons and disrupts axonal development, in a mechanism that likely involves glycinergic depolarization (Reynolds et al., 2008). These studies show critical roles for depolarizing GABAergic/glycinergic signaling in the generation of neuronal cell types.

Synaptogenesis is an intricate, multistep process that requires many cell intrinsic and extrinsic factors. Several studies have investigated the role of depolarizing GABA on synaptogenesis by manipulating the Cl^–^ gradient. For example, premature expression of Kcc2 in a subpopulation of ventricular progenitors and progenitor-derived cortical neurons leads to neurons with fewer and shorter dendrites, indicating that depolarizing GABAergic signaling mediates morphological maturation (Cancedda et al., 2007). In contrast, inhibition of Nkcc1 leads to decreased dendrite density and increased dendrite length in newly born cortical neurons (Wang and Kriegstein, 2008). In line with this, disruption of depolarizing GABA in the dentate gyrus of adult mouse hippocampus alters the morphology of newly generated granule neurons (Ge et al., 2006). Finally, GABA_*A*_R activation through depolarizing GABA is essential for synaptic inputs on newborn cortical neurons and in adult mouse granule neurons (Ge et al., 2006; Wang and Kriegstein, 2008).

Pioneering GABA signaling can also influence E/I balance in developing neural circuits, as a premature GABA polarity shift in Xenopus tectal neurons and rat cortical neurons leads to an increase of inhibitory inputs compared to excitatory inputs (Chudotvorova et al., 2005; Akerman and Cline, 2006); a similar mechanism occurs in the mammalian cortex (Wang and Kriegstein, 2008). Lastly, depolarizing GABAergic neurotransmission also drives calcium influx by relieving the voltage dependent Mg^2+^ block, thereby initiating intracellular signaling cascades needed for glutamatergic synapse development (Leinekugel et al., 1995; Akerman and Cline, 2006; Ben-Ari et al., 2007). In summary, depolarizing GABA influences the critical neurodevelopmental mechanisms of neurogenesis and synaptogenesis.

### Neural circuit formation

It is important to note that GABAergic currents often mature before glutamatergic counterparts, and GABA is the first and sole source of neurotransmission in various brain structures (Chen et al., 1996; Van Den Pol et al., 1998; Tyzio et al., 1999; Gao and Van Den Pol, 2001; Hennou et al., 2002; Gozlan and Ben-Ari, 2003; Johnson et al., 2003; Wang and Kriegstein, 2008). Therefore, the timing and degree of depolarizing GABAergic signaling in immature neurons is vital for the formation of neural networks. For example, GABAergic hub interneurons play essential roles in synchronizing hippocampal networks by orchestrating the generation of giant depolarizing potentials (GDP); GDP represent the first synapse-driven patterns of activity that help drive the synchronous network activity required for synaptic plasticity (Bonifazi et al., 2009; Picardo et al., 2011). Interestingly, stimulation of hippocampal hub neurons reduces network oscillations and in extreme cases stimulation completely desynchronizes activity (Bonifazi et al., 2009). While this data suggests considerable complexity in hub neuron physiology, it ultimately shows the influence on these pioneer GABAergic cells on the emergence of patterned activity in the brain.

The development of motor circuits in the spinal cord also appear to require synchronizing influence by GABAergic interneurons. GABA and glycine depolarize immature motor neurons in the rodent spinal cord (Wu et al., 1992); these neurotransmitters also drive spontaneous motor neuron activity (Nishimaru et al., 1996). In zebrafish, premature overexpression of Kcc2 reduces early locomotion, which suggests that early motor networks must form prior to the reversal of the chloride gradient (Reynolds et al., 2008). Moreover, GABAergic ventrolateral descending (VeLD) interneurons are highly active in early zebrafish embryogenesis and are physiologically coupled with other spinal neurons, including motor neurons; VeLD appear to act as central pattern generators in generating the earliest spontaneous movements, as inhibition of VeLD activity disrupts the integration of neurons into the motor network (Saint-Amant and Drapeau, 2000; Warp et al., 2012). Taken together, depolarizing activity from GABAergic neurons provides essential synchronizing influence on the emergence of motor circuits.

Foundational alterations in the formation of motor circuits in embryogenesis could have lasting consequences. Hyperexcitable motor behavior is a principal phenotype in various FXS models at many stages of development and maturity (Kim et al., 2014; Shamay-Ramot et al., 2015; Sørensen et al., 2015; Hu et al., 2020; Liu et al., 2022). Depolarizing GABAergic interneurons are crucial for the formation of neural circuits, and the GABAergic polarity shift is hindered in FXS. Importantly, GABA_*A*_R expression and GABA_*A*_R-mediated inhibition is actually reduced in FXS patients and models and is theorized to underlie hyperexcitable behavior (El Idrissi et al., 2005; D’Hulst et al., 2006; Morin-Parent et al., 2019). It is important to note that depolarizing glycinergic contributions to neurodevelopment are far less explored in FXS models and it is unclear if glycinergic signaling provides an influence that is distinct from GABA. However, altered glycinergic reception is linked to ASD in a mechanism that appears rooted in embryogenesis (Pilorge et al., 2016). Although it is difficult to link developmental roles for GABAergic/glycinergic signaling with later inhibitory requirements, new FXS models must integrate cellular and molecular approaches in embryonic stages with behavioral and physiological readouts in progressive developmental timepoints.

## Non-canonical roles for the chloride transporter Kcc2

In addition to ion transport function, Kcc2 also plays structural roles in the generation and function of dendritic spines, as Kcc2 mediates actin dynamics by interacting with β-PIX (β isoform of Rac/Cdc42 guanine nucleotide exchange factor), and with the aid of Rac1 GTPase controls the phosphorylation of Cofilin-1, an actin regulating protein (Chevy et al., 2015; Llano et al., 2015). Cofilin function includes the severing of actin filaments which leads to increased filament turnover, and cofilin-mediated action allows the insertion of AMPA receptors during chemically induced long-term potentiation (LTP) (Bamburg and Bernstein, 2010; Gu et al., 2010). Kcc2 also binds 4.1N, a cytoskeleton-associated protein that regulates lateral diffusion of both Kcc2 and AMPA receptors at excitatory synapses (Li et al., 2007; Gauvain et al., 2011; Chamma et al., 2013). Interestingly, Kcc2 overexpression or downregulation leads to changes in synaptogenesis, including dendritic spines that resemble those in patients with FXS and *Fmr1* KO mice (Irwin et al., 2001; Li et al., 2007; Fiumelli et al., 2013; Awad et al., 2018). This phenotype is speculated to contribute to FXS symptoms and severity, as modulation of actin cytoskeleton dynamics ameliorates seizures, rescues behavioral abnormalities (hyperactivity and repetitive movements), and reverses spine abnormalities displayed in *Fmr1* KO mice (Dolan et al., 2013). Kcc2 also physically interacts with a variety of membrane and scaffolding proteins, such as metabotropic GABA_B_ receptors; the inhibitory post-synaptic scaffold Gephyrin, required for clustering of GABAergic and glycinergic receptors at the postsynaptic membrane; the GluK2 and Neto2 subunits of Kainate glutamatergic receptors (KAR), implicated in both the kinetics and pre and postsynaptic modulation; and Task-3 (KCNK9) leak potassium channels, linked to network oscillations (Ivakine et al., 2013; Mahadevan et al., 2014; Wright et al., 2017; Goutierre et al., 2019; Kesaf et al., 2020; Al Awabdh et al., 2022). These non-canonical roles for Kcc2 establish the Cl^–^ exporter as a persistent and crucial regulator of neuronal morphology and synaptic function.

## Pharmacological approaches and future therapeutics

Persistent changes in excitatory/inhibitory balance in patients with FXS has led to pharmaceutical strategies to restore E/I balance through modulation of GABAergic or glutamatergic neurotransmission. Limited GABA modulator clinical trials (acamprosate, arbaclofen, riluzole/risperidone, and valproate), have shown mixed and inconclusive results, but include improved social behavior, hyperactivity, communication skills, compulsive symptoms, and irritability, with no serious adverse effects (Wu et al., 1992; Kaila, 1994; Dzhala et al., 2005; Hellings et al., 2005; Hollander et al., 2006; Kirsch, 2006; Paluszkiewicz et al., 2011; Guida et al., 2015; Moore et al., 2017; Schulte et al., 2018; He et al., 2019; Tang et al., 2019; Urbanska et al., 2019; Liu et al., 2022). In addition, some glutamatergic antagonists (riluzole, aripiprazole, memantine, phenobam, mavoglurant, and basimglurant) have shown promising results in clinical trials, especially regarding hyperactivity, irritability, and social deficits, though outcomes were highly variable in the treatment of core symptoms (Findling et al., 2014; Ichikawa et al., 2017; Wink et al., 2018; Hardan et al., 2019). Optimal therapeutics must address the complexity of Glu/GABA signaling, the heterogeneity of ASDs, and how ASD pathophysiology varies across age (Masi et al., 2017; Ure et al., 2018).

Alternative pharmaceutical approaches have directly targeted chloride homeostasis to restore E/I balance *via* drugs targeted to Nkcc1 or Kcc2. In multiple FXS and ASD animal models the Nkcc1 inhibitor bumetanide rescues GABAergic neurotransmission as well as social and repetitive behaviors (Tyzio et al., 2014; Amin et al., 2017; Zeidler et al., 2017; Savardi et al., 2020). As mentioned previously, bumetanide also rescues somatosensory deficits and morphological changes in the Fmr1 knockout mouse (He et al., 2019). A recent clinical trial on bumetanide in children and adolescents with ASD showed promising results by improving symptoms of ASD; however, several limitations such as short observation periods, lack of older ASD participants, and exclusion of participants with comorbidities (epilepsy) should be considered (Lemonnier et al., 2017). Other clinical trials with bumetanide have produced mixed results, which may be due to the blood-brain barrier, diuretic effects, and/or ototoxicity in infants (likely reflecting the ubiquitous expression of Nkcc1) (Löscher et al., 2013; Ben-Ari et al., 2016; Römermann et al., 2017; Nussbaum and Perazella, 2019; Crutel et al., 2021). Therefore, further testing is needed to determine efficient and safe dosages for chronic treatment. Accordingly, additional bumetanide prodrugs and analogs (BUM1, BUM5, bumepamine, azosemide, STS66, BUM9, ARN23746) have been developed to optimize selectivity and brain penetration (Töllner et al., 2014; Erker et al., 2016; Brandt et al., 2018; Hampel et al., 2018; Huang et al., 2019; Savardi et al., 2020).

In related NDDs, additional approaches to minimize hyperexcitable circuit function have focused on enhancement of the chloride exporter Kcc2. These pharmacological approaches include FDA approved Kcc2 expression-enhancing compounds such as BIO (inhibitor of GSK3b), KW-2449 (inhibitor of Flit3), piperine (agonist of TRPV1), and resveratrol (activator of SIRT1) (Guida et al., 2015; Tang et al., 2019; Urbanska et al., 2019). For example, KW-2449 and BIO can hyperpolarize neurons cultured from human Rett Syndrome patients to values comparable to wild type, and restore morphology (Tang et al., 2019). In addition, treatment of Mecp2 knockout mice (a model of Rett syndrome) with KW-2449 or piperine rescues respiratory and locomotion phenotypes (Tang et al., 2019). These results in Rett Syndrome models may represent promise for FXS and other NDDs that also display altered Cl^–^ transporter expression. However, more studies are needed to elucidate the mechanisms of action and potential side effects, and approaches to promote Kcc2 expression and function must also include membrane targeting to achieve transporter function. Although these approaches are in infancy, this neuron-specific transporter has perhaps the most promise as a pharmaceutical target, as Kcc2 function can directly minimize chloride content and reduce hyperexcitability.

## Discussion

What is the link between FMRP and chloride balance? Changes in chloride homeostasis in developing neurons underlies the fascinating polarity shift that occurs in the maturation of inhibitory circuits; this maturation is delayed in FXS, which may have foundational consequences on the formation and function of the nervous system. We speculate that in the absence of FMRP, deficient neuronal differentiation leaves immature neurons locked in an elevated chloride state, and thus more prone to excitation. There are several clues pointing to this mechanism: 1) Nkcc1 inclusion and Kcc2 exclusion from precursor cells (Li et al., 2002; Wang et al., 2002; Stein et al., 2004); 2) elevated Nkcc1 expression in FXS knockout mice (He et al., 2014); 3) deficient differentiation in the absence of FMRP, as seen in neural precursor cells and spinal cell subtypes (Luo et al., 2010; Edens et al., 2019; Doll et al., 2021; Raj et al., 2021). As FMRP appears to bind both *Slc12a2* and *Slc12a5* (the genes encoding Nkcc1 and Kcc2) (Darnell et al., 2011), there could be a direct regulatory role for the RNA binding protein in the transport/localization, stability, or translation of these transcripts. The mechanism could also be indirect through microRNA-based repression of Nkcc1 (Liu et al., 2015; Lippi et al., 2016). Given regional and temporal differences in transporter expression during development, rescuing chloride imbalance on a global level will be especially challenging.

Is there a link or point of convergence between Cl^–^ transporter function and disparate NDDs? Although a diverse family of NDDs (FXS, Rett Syndrome, DiGeorge syndrome, Dravet syndrome, epilepsy, etc.) are linked to unique genetic loci, they have common symptoms and comorbidities and are associated with altered Cl^–^ transporter expression. In other words, changes in the crucial neurodevelopmental polarity shift are common to disease states with vast genetic heterogeneity. Given these commonalities across NDDs, we predict that the progression of transporter expression is linked with neuronal maturation, such that alterations in neurogenesis and differentiation in unique disease states lie upstream of chloride homeostasis. Although this suggests independent genetic mechanisms underlie chloride balance, it also grants broader therapeutic opportunities through refined drugs targeting these transporters.

Is there a link between depolarizing GABAergic/glycinergic function in embryogenesis to deficient inhibition at maturity? Following the neuro-archeology concept formulated by Ben-Ari, if synchronizing GABAergic neurons maintain a persistent depolarizing influence they could drive extended immature currents and oscillations in the developing brain that would ultimately perturb the developmental sequence and operation of functional networks (Ben-Ari, 2008, 2014). We speculate that this could present runaway hyperexcitability in the form of cognitive impairment and seizure susceptibility shown in neurodevelopmental disabilities and various forms of epilepsy (Giachello and Baines, 2015). Neural circuits develop hierarchically, within narrow critical periods that shape connectivity and long-term function (Hensch, 2004; LeBlanc and Fagiolini, 2011). Although many of these critical periods have been dissected through elegant developmental studies, it is still very difficult to link early neurodevelopmental events (neurogenesis, fate specification, cell migration, etc.) with long term functional output (behavior). The restoration of E/I balance represents a main tenet of FXS and ASD modeling and entails either a restoration of inhibition or a dampening of excitation. While many studies have shown deficient GABAergic signaling in established FXS circuits, roles for FMRP in early development, prior to the polarity shift, are less explored.

## Data availability statement

The original contributions presented in this study are included in the article/supplementary material, further inquiries can be directed to the corresponding author.

## Author contributions

KM: research and writing. CD: editing and visualization. Both authors contributed to the article and approved the submitted version.
